# Something is amiss in Denmark: A comparison of preventable hospitalisations and readmissions for chronic medical conditions in the Danish Healthcare system and Kaiser Permanente

**DOI:** 10.1186/1472-6963-11-347

**Published:** 2011-12-22

**Authors:** Michaela Schiøtz, Mary Price, Anne Frølich, Jes Søgaard, Jette K Kristensen, Allan Krasnik, Murray N Ross, Finn Diderichsen, John Hsu

**Affiliations:** 1 Steno Health Promotion Center, Steno Diabetes Center, Niels Steensensvej 8, DK-2820 Gentofte, Denmark; 2 Section for Health Services Research, Department of Public Health, Faculty of Health Science, University of Copenhagen, Øster Farimagsgade 5, Building 10, DK-1014 Copenhagen K, Denmark; 3 Division of Research, Kaiser Permanente Medical Care Program, 2000 Broadway, Oakland, CA 94612, USA; 4 Mongan Institute for Health Policy, Massachusetts General Hospital and Partners Health Care System, 50 Staniford Street, 9thFloor, Boston, MA, 02114; Department of Health Care Policy, Harvard Medical School, 180 Longwood Ave, Boston, MA, 02115, USA; 5 Copenhagen Hospital Cooperation, Bispebjerg Bakke 23, Bispebjerg Hospital; DK-2400 Copenhagen NV, Denmark; 6 Danish Institute for Health Services Research, Dampfærgevej 27-29, DK-2100 Copenhagen Ø, Denmark; 7 Department of General Practice, University of Aarhus, Bartholins Allé 2, DK-8000 Aarhus, Denmark; 8 Kaiser Permanente Institute for Health Policy, One Kaiser Plaza, 22nd Floor, Oakland CA 94612 USA

## Abstract

**Background:**

As many other European healthcare systems the Danish healthcare system (DHS) has targeted chronic condition care in its reform efforts. Benchmarking is a valuable tool to identify areas for improvement. Prior work indicates that chronic care coordination is poor in the DHS, especially in comparison with care in Kaiser Permanente (KP), an integrated delivery system based in the United States. We investigated population rates of hospitalisation and readmission rates for ambulatory care sensitive, chronic medical conditions in the two systems.

**Methods:**

Using a historical cohort study design, age and gender adjusted population rates of hospitalisations for angina, heart failure, chronic obstructive pulmonary disease, and hypertension, plus rates of 30-day readmission and mortality were investigated for all individuals aged 65+ in the DHS and KP.

**Results:**

DHS had substantially higher rates of hospitalisations, readmissions, and mean lengths of stay per hospitalisation, than KP had. For example, the adjusted angina hospitalisation rates in 2007 for the DHS and KP respectively were 1.01/100 persons (95%CI: 0.98-1.03) vs. 0.11/100 persons (95%CI: 0.10-0.13/100 persons); 21.6% vs. 9.9% readmission within 30 days (OR = 2.53; 95% CI: 1.84-3.47); and mean length of stay was 2.52 vs. 1.80 hospital days. Mortality up through 30 days post-discharge was not consistently different in the two systems.

**Conclusions:**

There are substantial differences between the DHS and KP in the rates of preventable hospitalisations and subsequent readmissions associated with chronic conditions, which suggest much opportunity for improvement within the Danish healthcare system. Reductions in hospitalisations also could improve patient welfare and free considerable resources for use towards preventing disease exacerbations. These conclusions may also apply for similar public systems such as the US Medicare system, the NHS and other systems striving to improve the integration of care for persons with chronic conditions.

## Background

Healthcare systems are undergoing reform in many countries including England, the United States, and Denmark. In all countries, there is interest in improving the quality and efficiency of medical care, as well as heightened concern about the continued growth in medical spending both in absolute terms and relative to other sectors of the economy, e.g., spending as a percentage of each nation's Gross Domestic Product. As more nations and systems within each nation strive to improve care, there is need for comparative information, at a minimum to provide benchmarks and examples of what is possible [1-3].

Much attention has focused on hospital care, which is particularly costly for both society and individual patients. For patients with chronic medical conditions, hospitalisations are easily measureable clinical events that represent incident cases or exacerbations of the underlying condition. Previous research also suggests that these events can be sensitive to the quality and amount of prior care, thus the rates of hospitalisations or readmissions when there is an initial hospitalisation, provide information on medical quality, including on the level of care coordination for patients with these complex conditions [4-10]. High numbers of hospitalisations for ambulatory care sensitive conditions (ACSCs) have been identified in a number of European countries, as well as among subpopulations in the United States with known problems with care such as patients without healthcare insurance or who receive care in highly fragmented systems [11-14].

Within recent years the US managed care organisation Kaiser Permanente (KP) has started to influence the mindsets and policy development within many European healthcare systems [15]. KP has been highlighted as a successful model of integrated and cost-effective care and prior work has found that the Northern California Region of KP had fewer hospital admissions for chronic diseases compared to the National Health Service (NHS) [16]. More recent research have indicated substantially poorer care coordination during transitions from hospital to primary care providers in the Danish Healthcare System (DHS), compared to KP [17].

We hypothesised that KP had lower rates of hospitalisation and readmissions given hospitalisation for preventable hospitalisations for chronic medical conditions compared to the DHS. Our larger immediate objective was to investigate the potential areas for chronic care improvement in the DHS, including basic estimates of excess hospitalisations within the Danish system relative to the Kaiser benchmark.

## Methods

### Settings and population

The two settings have several substantial differences in the organisation and reimbursement of care. In past studies, KP has been highlighted as a successful model of integrated care with a strong focus on prevention, primary care facilities organised in highly specialised medical centres employing both primary care and speciality care physicians, and well developed, integrated health information management systems [18,19]. In the DHS, outpatient care entails self-employed general practitioners (GPs) serving as "gatekeepers" to the healthcare system and specialists working in private practice and at in ambulatory clinics located within hospitals. While the DHS also has extensive use of computer-based clinical systems, these systems are typically not integrated, and clinical information is rarely shared electronically between GPs and specialists or hospitals.

KP operates in nine states and Washington DC and is the largest not-for-profit managed care organization in the US with 8.2 million members [20]. KP is a consortium of three separate but interdependent groups of entities: the Kaiser Foundation Health Plan and its regional operating organizations, Kaiser Foundation Hospital and the Permanente Medical Groups. Kaiser Foundation Health Plan and Hospitals are integrated with legal separate physician group practices called Permanente Medical Groups. The health plan is the insurance part of the organisation, while the hospitals and medical group provide all clinical services. KP is financed from membership premiums and co-payments pooled by Kaiser Foundation and reallocated to the medical centres and tightly linked multi-specialty physician group according to annual contracts. All physicians in The Permanente Medical Group (TPMG) are reimbursed by salary; Kaiser Foundation hospitals operate under annual budgets. Within KP, comprehensive health services are provided including hospital admission, sub-acute care, ambulatory and preventive care, accident and emergency, optometry, rehabilitation, and home healthcare [21]. A typical patient in need of primary care, e.g. due to a chronic condition, will, in KP be treated and cared for solely in an out-patient medical centre. The medical centre will have all necessary outpatient facilities available, including internal medicine physicians, geriatricians, specialists, nurse practitioners, nurses, health educators, administrative personnel, a pharmacy, and an emergency department. The physicians have access to in-house laboratory facilities and other advanced medical equipment. When necessary, patients are admitted to a hospital, and subsequent care and some rehabilitation will be administered outside the hospital at a skilled nursing facility (SNIF). Information exchange between providers is facilitated through the operational electronic health record KP HealthConnect. This system also allows for multiple patient panel management and two way patient contact [22].

The DHS is funded mainly through taxation and belongs to the same family of healthcare systems as those of the other Scandinavian countries and the United Kingdom [23,24]. The DHS covers all inhabitants. Danish health services are delivered partly by small physician practices and partly public hospitals. Specific public health services are delivered by the municipalities. Physician practices include both GP practices and specialist practices, the latter co-providing out-patient services with the hospitals. They are owned and run by the senior physicians according to contracts with the Public Health Insurance. GP services are financed by a mixture of fee for service (75%) and capitation (25%) and specialist services entirely by fee for services. Hospitals are reimbursed by a combination of budgets and DRGs per admissions. Since 2004, 50% of the hospitals income must be DRG value based. Prior to 2007 the health services were delivered by 14 counties headed by elected politicians; public expenditure for the Public Health Insurance, of the public hospitals and fees for patients admitted to private hospitals is financed by proportional income county-taxes (65%) and national grants. Since 2007 the same services are delivered by five regions headed by elected politicians and the public expenditure is financed by a demographically adjusted national grant (80%) and municipality payments (20%) [23].

A prior study comparing inputs and performance of KP and the DHS shows that KP employs fewer physicians per capita than the DHS and has a lower use of hospital beds and lower acute care admission rates than the DHS. However, per capita expenditures are significantly higher for KP than the DHS, in part because of salary differences for clinical staff as well as differences in the numbers and types of support staff. The same study showed that more KP members reported having documented chronic medical conditions than did Danish citizens: 6.3% reported having diabetes mellitus in KP vs. 2.8% in DHS; 19% reported having hypertension in KP vs. 8.5% in DHS; and 1.0% reported having a stroke in KP vs. 0.2% in DHS [25].

In this study, we focused on people aged 65 and over to increase the comparability of the two populations. For example, in the US, citizens aged 65 years and older are eligible for publicly financed insurance coverage (Medicare) at levels comparable to the DHS. Other ongoing work by our group suggests that the Kaiser Medicare population also resembles the overall US Medicare population, at least with respect to predicted Medicare spending.

### Measures, data sources, and data collection

We used the definition of ambulatory care sensitive conditions (ACSCs) from the U.S. Agency for Healthcare Research and Quality (AHRQ), which defines them as admissions for diagnoses that could have been prevented or ameliorated with currently recommended outpatient care, according to recent evidence from population-based studies [26]. The face validity, precision, minimum bias, and construct validity have been described elsewhere [27]. In this report, we focus on hospitalisations for ACSCs for five selected chronic medical conditions: angina (without procedures), chronic obstructive pulmonary disease (COPD), congestive heart failure (CHF), diabetes mellitus (DM), and hypertension (HTN). We used the population sizes of individuals aged 65+, in each system in each year, to calculate the rates of these clinical events representing chronic condition exacerbations. In other words, for both the DHS and KP, we divided the numbers of subjects with each of the five types of clinical events by the total number of subjects alive in January of each year. To see how hospitalisation rates compared for a non-preventable, non-elective admission in the two systems, we also examined rates of appendicitis, which almost always requires a hospitalisation, are unlikely to be related to quality of outpatient medical care, and provides information on potential differences in access to care [28].

We translated ICD-9 diagnosis codes used in the AHRQ specifications of ACSCs to ICD-10 codes using the New Zealand Health Information Service ICD-10/ICD-9 converter http://www.nzhis.govt.nz/moh.nsf/pagesns/254. Subsequently, two co-authors with clinical backgrounds reviewed the codes to improve sensitivity and to ensure that the ICD-10 codes covered the ICD-9 codes used in the AHRQ definition (see additional file 1). Further, leading hospital specialists in the DHS were contacted in order to validate that the ICD-10 codes covered the codes used in practice. In keeping with the AHRQ definition, we excluded transfers from other hospital departments (except transfers from emergency departments, outpatient clinics, and transfers from outside the KP system).

We obtained data for 2002 through 2007 on length of stay, principal diagnosis, hospitalisation date, hospital and department, and type of contact to the hospital (admission, outpatient visit, short-stay admission, or emergency department visit). The Danish National Patient Registry and KP's administrative and clinical databases provided information about emergency department visits, ambulatory visits, hospitalisations with principal diagnosis, age, and gender.

We also examined deaths among these patients with chronic conditions. While death arguably will be influenced by both medical and non-medical factors including genetic, lifestyle, and socio-economic conditions, compared with hospitalisations, findings of lower hospitalisations combined with greater mortality would raise concerns about underuse of medical care. Similarly, findings of shorter lengths of stay combined with greater mortality would raise concerns about premature hospital discharge. We obtained mortality data from the Danish Civil Registration System, and from KP's administrative and clinical databases. We obtained information about population size from Statistic Denmark and KP's administrative databases. The Kaiser Foundation Research Institute Institutional Review Board reviewed and approved the study. Separate Danish ethics approval was not required.

### Statistical analysis

We calculated annual ACSC hospitalisation rates for the five selected conditions in 2002-2007. Using the population sizes by age and gender in January of each year as the denominator, we calculated age- and gender-specific hospitalisation rates for each system in each year. We used direct standardisation to find the annual age- and gender- adjusted rates for each of the five conditions, taking differences in the structure of the two populations into account by standardising the KP population to the Danish population. The same analyses were repeated, excluding one-day hospitalisations to investigate if they were used instead of outpatient visits in one system and not the other. We used a logistic model to calculate the odds of rehospitalisation within 30 days after discharge for persons hospitalised with an ACSC for one of the five selected conditions in each year. The models only include patients discharged alive and adjusted for age, gender and month of initial admission. We calculated length of stay for all ACSC hospitalisations excluding in-patients deaths for the five selected conditions, and the odds of death up to 30 days after discharge. In sensitivity analyses, we separately examined the odds of death during the hospitalisation, and the odds of death within 30 days of the initial hospitalisation for an ACSC for one of the five selected conditions, again using logistic models adjusted for age, gender, length of stay, and year and month of admission.

In order to estimate the approximate resources associated with the higher rehospitalisation rates in the DHS, we used Diagnosis Related Group (DRG) costs obtained from the Danish National Patient Registry. This estimation of the DHS costs if it had event rates equivalent to those in KP provide an upper bound on the resources potentially available for reallocation in the DHS.

## Results

In 2002, there were 794,575 persons in the DHS and 374,290 persons in KP who were 65+ years of age; and in 2007, there were 834,741 and 399,270 persons in each system respectively. During this period between 2002 and 2007, 159,322 in the DHS and 32,710 in KP were hospitalised at least once for exacerbations of one of the five ambulatory care sensitive, chronic medical conditions.

### Hospitalisation rates for chronic medical conditions

The hospitalisation rates in DHS decreased significantly between 2002 and 2007: there was a 53% reduction in hospitalisations for angina in DHS between 2002 and 2007; 38% reduction for COPD; 46% reduction for heart failure, 41% reduction for diabetes; and 13% reduction for hypertension.

Despite the large DHS reductions over this six year period, the rates of hospitalisation remained significantly higher in the DHS compared to KP (Table 1). For all five conditions together, the 2007 age- and gender-standardised hospitalisation rates were 2.5 times higher in the DHS compared with KP: 5.21 hospitalisations/100 persons (95%CI: 5.17-5.26) and 2.02 hospitalisations/100 persons (95%CI: 1.98-2.06) in DHS and KP respectively. Across conditions, the differences in hospitalisation rates ranged from 9.2 times greater in DHS compared to KP for angina to 1.1 times greater for CHF: for angina, the rate was 1.01 hospitalisations/100 persons in the DHS, 95%CI:0.98-1.03, compared with 0.11 hospitalisations/100 persons in KP, 95%CI:0.10-0.13; and for CHF, the rate was 0.91 hospitalisations/100 persons 95%CI: 0.89-0.93, compared with 0.85 hospitalisations/100 persons 95%CI: 0.82-0.88.

**Table 1:** Hospitalisation rates per 100 persons aged 65 and over and mean length of stay (LOS)*

		DHS				KP			
		Rate	(95% CI)	LOS	(SD)	Rate	(95% CI)	LOS	(SD)
Angina	2002	2.14	(2.11 - 2.17)	2.60	(3.4)	0.15	(0.14 - 0.17)	2.37	(2.0)
	2003	1.96	(1.93 - 1.99)	2.46	(3.4)	0.13	(0.11 - 0.14)	2.07	(1.6)
	2004	1.38	(1.35 - 1.40)	2.45	(3.1)	0.12	(0.11 - 0.13)	1.92	(1.5)
	2005	1.30	(1.28 - 1.33)	2.31	(3.0)	0.11	(0.10 - 0.12)	1.83	(1.3)
	2006	1.16	(1.14 - 1.19)	2.54	(3.1)	0.11	(0.10 - 0.12)	1.85	(1.3)
	2007	1.01	(0.98 - 1.03)	2.52	(3.3)	0.11	(0.10 - 0.13)	1.80	(1.3)

COPD	2002	3.02	(2.98 - 3.06)	4.74	(6.3)	0.73	(0.70 - 0.76)	4.15	(5.2)
	2003	2.88	(2.84 - 2.92)	4.42	(5.7)	0.68	(0.65 - 0.71)	4.19	(4.8)
	2004	2.19	(2.16 - 2.22)	4.35	(5.7)	0.61	(0.58 - 0.63)	3.77	(4.2)
	2005	2.10	(2.06 - 2.13)	4.36	(6.8)	0.58	(0.55 - 0.60)	3.69	(3.9)
	2006	2.07	(2.04 - 2.10)	4.18	(6.0)	0.53	(0.50 - 0.55)	3.94	(3.9)
	2007	1.86	(1.84 - 1.89)	3.98	(5.8)	0.50	(0.48 - 0.52)	4.03	(6.6)

CHF	2002	1.70	(1.68 - 1.73)	6.02	(8.1)	1.45	(1.41 - 1.49)	3.95	(4.0)
	2003	1.65	(1.62 - 1.68)	5.94	(14.8)	1.16	(1.12 - 1.19)	3.84	(3.8)
	2004	1.11	(1.09 - 1.13)	6.16	(6.9)	1.19	(1.16 - 1.23)	3.87	(4.2)
	2005	1.00	(0.98 - 1.02)	5.94	(6.7)	1.09	(1.06 - 1.13)	4.05	(4.7)
	2006	0.97	(0.95 - 0.99)	6.12	(7.1)	0.93	(0.90 - 0.96)	4.45	(5.2)
	2007	0.91	(0.89 - 0.93)	5.68	(5.9)	0.85	(0.82 - 0.88)	4.27	(4.2)

Diabetes	2002	1.28	(1.26 - 1.31)	6.84	(12.9)	0.34	(0.32 - 0.36)	4.43	(6.6)
	2003	1.38	(1.35 - 1.40)	7.08	(13.6)	0.37	(0.35 - 0.39)	4.40	(7.1)
	2004	0.98	(0.96 - 1.00)	7.05	(9.1)	0.41	(0.39 - 0.43)	4.04	(5.1)
	2005	0.90	(0.88 - 0.93)	6.14	(8.4)	0.46	(0.44 - 0.48)	4.60	(7.0)
	2006	0.97	(0.95 - 0.99)	6.22	(6.8)	0.45	(0.43 - 0.47)	4.20	(6.0)
	2007	0.75	(0.73 - 0.77)	5.62	(6.8)	0.48	(0.46 - 0.49)	3.96	(6.2)

Hypertension	2002	0.80	(0.78 - 0.82)	3.56	(8.2)	0.06	(0.05 - 0.06)	2.18	(1.9)
	2003	0.88	(0.86 - 0.90)	3.68	(5.4)	0.06	(0.05 - 0.07)	2.09	(2.1)
	2004	0.69	(0.67 - 0.71)	3.50	(6.7)	0.07	(0.06 - 0.08)	2.15	(2.6)
	2005	0.66	(0.63 - 0.67)	3.33	(5.1)	0.06	(0.05 - 0.07)	1.98	(1.8)
	2006	0.72	(0.70 - 0.74)	3.22	(4.3)	0.08	(0.07 - 0.08)	2.03	(1.8)
	2007	0.70	(0.68 - 0.71)	3.04	(3.6)	0.08	(0.07 - 0.09)	2.38	(2.9)

*Age and gender adjusted hospitalisation rates and Mean Length of Stay (LOS) for all hospitalisations with the given diagnosis excluding inpatients deaths

In sensitivity analyses that excluded one-day hospitalisations, the differences in hospitalisation rates between DHS and KP for all five chronic conditions were similar to the analyses using all hospitalisations. In contrast, there was no statistically significant difference between the hospitalisation rates for appendicitis in the DHS and KP between 2002 and 2007 together. The rate was 0.07/100 persons in the DHS, 95%CI: 0.06-0.07 compared with 0.06/100 persons in KP, 95%CI: 0.06-0.07.

For all conditions, the mean length of stay was greater in DHS compared with KP, though the mean decreased significantly in DHS between 2002 and 2007 (for all five conditions, the mean LOS was 4.63 days in 2002 and 4.08 days in 2007; in KP, the mean LOS was 3.94 days in 2002 and 3.91 days in 2007.

### Readmissions

In addition to the higher initial hospitalisation rates in the DHS compared to KP, the percentage of patients having a readmission also was higher in the DHS compared to KP, e.g., 21.0% vs. 19.5% readmission (OR = 1.10 for DHS vs. KP, 95%CI:1.03-1.16), for all five conditions in 2007 (Table 2). The largest difference in readmission rates between the two systems was for readmissions after an initial hospitalisation for angina, e.g., 23.0% vs. 12.4% in 2002 (OR = 2.12, 95% CI: (1.64-2.74)) and 21.6% vs. 9.9% in 2007 (OR = 2.53, 95% CI:(1.84-3.47)) in the DHS and KP respectively. In contrast, the odds of being readmitted within 30 days after a hospitalisation for diabetes were significantly higher in KP than in the DHS. The odds of being readmitted after being hospitalised with COPD, hypertension, or CHF did not differ substantially between the two systems for the majority of the study period.

**Table 2:** Percentage of people aged 65 and over readmitted within 30 days after a hospitalisation^a^

	2002			2003			2004			2005			2006			2007		
	**DHS** **%**	**KP** **%**	**OR** **(95% CI)**	**DHS** **%**	**KP** **%**	**OR** **(95% CI)**	**DHS** **%**	**KP** **%**	**OR** **(95% CI)**	**DHS** **%**	**KP** **%**	**OR** **(95% CI)**	**DHS** **%**	**KP** **%**	**OR** **(95% CI)**	**DHS** **%**	**KP** **%**	**OR** **(95% CI)**

Angina	23.0	12.4	2.12 (1.64-2.74)	22.9	13.6	1.88 (1.45-2.44)	22.8	9.3	2.87 (2.07-3.86)	25.1	10.6	2.82 (2.07-3.86)	23.0	10.1	2.67 (1.94-3.67)	21.6	9.9	2.53 (1.84-3.47)

COPD	24.2	20.1	1.27 (1.15-1.41)	24.1	19.4	1.32 (1.19-1.46)	24.1	20.4	1.25 (1.12-1.39)	23.9	20.3	1.23 (1.10-1.38)	22.6	20.7	1.12 (1.00-1.26)	23.2	21.4	1.11 (0.99-1.25)

CHF	24.0	21.7	1.14 (1.05-1.24)	23.7	21.3	1.15 (1.05-1.25)	23.4	22.2	1.07 (0.98-1.18)	24.4	23.3	1.06 (0.97-1.16)	23.0	23.0	0.99 (0.90-1.10)	24.1	21.4	1.17 (1.05-1.29)

Diabetes	18.0	20.1	0.87 (0.75-1.02)	16.4	20.6	0.75 (0.66-0.86)	15.3	21.2	0.67 (0.59-0.77)	16.5	22.0	0.70 (0.61-0.79)	14.3	21.3	0.62 (0.54-0.70)	16.1	18.4	0.85 (0.74-0.98)

Hypertension	16.4	14.2	1.18 (0.79-1.78)	17.1	9.2	2.04 (1.30-3.22)	15.7	10.0	1.68 (1.12-2.52)	16.9	13.4	1.32 (0.89-1.94)	15.8	11.7	1.41 (0.98-2.03)	15.3	13.4	1.17 (0.84-1.64)

^a ^Adjusted for age, gender, and month of initial hospitalisation

### Mortality

Table 3 displays the percentage of hospitalisations resulting in death either during the hospitalisation or up to 30 days after discharge after a hospitalisation for each of the five chronic medical conditions, as well as the percent dying during the hospitalisation and after discharge. The difference in mortality varied across the five conditions, with statistically significant higher odds of dying in DHS among patients admitted for heart failure compared with KP, significantly lower odds of dying in DHS compared with KP among patients admitted for COPD, and no significant differences among the other three conditions.

**Table 3:** Percentage of ACSC hospitalisations ending in death during and within 30 days of discharge^a^

	All deaths during and within 30 days of hospitalisation			Deaths during initial hospitalization			Deaths within 30 days of discharge		
	**DHS** **%**	**KP** **%**	**OR** **(95% CI)**	**DHS** **%**	**KP** **%**	**OR** **(95% CI)**	**DHS** **%**	**KP** **%**	**OR** **(95% CI)**

Angina	2.3	1.8	1.27 (0.96-1.68)	0.4	0.2	1.77 (0.83-3.75)	1.9	1.6	1.19 (0.88-1.61)

COPD	8.5	10.5	0.79 (0.74-0.83)	2.6	2.8	0.94 (0.85-1.04)	5.9	7.7	0.74 (0.69-0.79)

CHF	18.0	13.1	1.47 (1.40-1.53)	8.6	3.6	2.51 (2.34-2.70)	9.5	9.5	1.00 (0.95-1.05)

Diabetes	5.8	5.9	0.97 (0.89-1.07)	2.1	1.3	1.60 (1.33-1.92)	3.7	4.6	0.79 (0.71-0.88)

Hypertension	1.3	1.1	1.20 (0.76-1.89)	0.5	0.2	2.05 (0.76-5.54)	0.9	0.9	0.99 (0.59-1.64)

OR = Odds ratio

^a^Adjusted for age, gender and year and month of admission

## Discussion

The initial hospitalisation and mean length of stay for five ambulatory care sensitive chronic medical conditions was substantially higher on average in the Danish healthcare system, compared with the Kaiser Permanente Integrated Delivery System. Subsequent readmission rates also were higher in the DHS for angina compared to the KP benchmark, but lower for diabetes compared to KP. The findings on mortality were mixed. There was a higher mortality in the DHS for patients with heart failure and lower mortality for chronic obstructive pulmonary disease, but no statistically significant differences for the other three conditions. In short, patients in the Danish healthcare system appear more likely to require preventable hospitalisations associated with chronic medical conditions, and have longer hospitalisations on average, compared with patients in the KP system.

There are several strengths of the study, including the quite large populations observed and the multi-year timeframe; our adjustment of analyses for differences in population characteristics and the timing of hospitalisations; and our inclusion of a condition that is not sensitive to ambulatory care as a control. Other strengths include the focus on two health systems with excellent capture of hospital data within electronic databases.

Comparing clinical outcomes across systems, however, is challenging, even for relatively straightforward hospital events and survival. We used hospitalization rates for selected ACSC as an indicator for the quality of primary care to patients with chronic conditions. However, there are a number of potential alternative explanations for differences in hospitalisation rates between the two systems. These include unmeasured differences in health and culture of the populations of the two healthcare systems, the level and quality of data capture, variations in access to primary care due to formal and informal barriers, and practice patterns within each system. In several cases, we would expect a bias towards finding no differences in our outcomes; in other cases, the effects are difficult to predict. The main threat to the findings is potential different use of hospitals for equal diagnoses in the two systems.

Unmeasured differences in the clinical and socio-demographic characteristics between the two populations almost certainly contribute to some of the observed findings. The DHS sample consists of the entire Danish population aged 65 and older whereas the KP population is a non-random sample of the US population that is enrolled within a single health plan. Our ongoing work, however, suggests that the KP population closely resembles the US population aged 65+ with respect to predicted risk. The US population in general also may have larger range in income levels compared to the Danish population where the progressive taxation policy means that few are very rich and few are very poor. Unmeasured differences in the available public and private social services also could contribute to some of the observed findings. Public transportation, public food services and other public funded services are available in Denmark whereas such services are private in the US. More public services available could favor lower readmissions in the DHS compared to KP. However, it is difficult to predict how differences in clinical and socio-demographic population characteristics overall affect the outcomes because of counterbalancing forces.

There are a number of social contextual issues that could influence the results and for which there is an extensive literature. Factors associated with genes, cultural norms around diet and exercise, and individual behavior such as smoking all could contribute to population level differences in disease prevalence, disease severity, and numbers and types of co-morbid diseases. The direction of such factors, however, is difficult to predict. For example, prior studies showed that Danes tend to both exercise and smoke more that the KP population and the KP population has higher self-reported prevalence of chronic conditions than the Danish population [25].

Differences in data capture among the systems may also to some degree contribute to some of the differences between the healthcare systems. Diagnoses may be recorded differently between the systems, which would affect our findings. The intensity of diagnosis might also differ, though the direction of the net effect is unclear with more hospital use in the DHS resulting in greater opportunity for hospital related diagnoses, but potentially higher outpatient diagnostic intensity in the United States [29].

Another potential explanation for the differences in hospitalization rates between the two systems is different use of hospitals. Danish hospitals have a high number of beds available compared to hospitals in KP, and supply is a powerful determinant of utilisation, which also could lower admission thresholds [25,30]. Correspondingly, the two systems could have different thresholds for admission to- and discharge from the hospital. Differences in admission thresholds alone, however, should result in lower deaths in the system with more initial hospitalisations. Higher hospitalisation rates combined with lower death rates in a healthcare system might suggest potential inefficiency either because of unnecessary hospitalisations or hospitalisations preventable with earlier intervention in the outpatient setting. Higher hospitalisation rates combined with higher death rates in a healthcare system, however, might suggest more serious quality problems.

Subsequent readmission rates were higher in the Danish healthcare system for angina compared to the KP benchmark, whereas the readmission rates for diabetes were lower in the DHS compared to KP. There is amount of literature stating that readmission rates may not serve as a valid indicator for quality of care. E.g. a report by Williams and Fitton state that readmission, perhaps on several occasions, may be generally preferred to permanent admission, both by the patient and by the system [31] and other studies have identified associations between readmissions and underlying physical conditions [32,33]. We were not able to adjust the analyses for underlying chronic conditions as the data did not include enough secondary diagnoses to do proper case-mix adjustment. Consequently, differences in readmission rates between the systems may not be a good indicator of quality differences among the systems.

Factoring in all of these issues, we believe that our results are consistent with other studies suggesting problems in the quality of chronic disease care within the DHS. This prior work includes findings of a more comprehensive and systematic approach to disease detection and disease prevention in KP compared to the DHS. KP provides more medical (secondary and tertiary) prevention to its members and more self-management support is provided in KP compared to the DHS. Additionally, disease treatment and complication prevention within the healthcare systems will affect hospitalisation rates. The KP system has structured chronic care management programmes that integrate multiple elements, such as clinical guidelines, disease registries, proactive outreach, reminders, multidisciplinary care teams, and performance feedback to providers [34]. Also, KP's integrated IT system, the medical centers in KP housing GPs and specialists as well as aligned financial and non-financial incentives throughout the system in KP make the interactions between providers easier, leading to better coordination and more follow-up which we believe result in lower initial hospitalisation rates and lower readmission rates.

Programmes to improve chronic care management have only recently been introduced in the DHS and are still in the implementation phase and were not widespread when the data from this study was obtained. Additionally, the DHS is a more fragmented system with general practitioners, hospitals, and preventive and rehabilitation services being paid from different public sectors, without aligned incentives or a proactive approach to prevention. Thus, prior studies conducted in the DHS have indicated that lack of acute services in the municipalities responsible for home nursing care and nursing homes to some extent caused undesirable hospitalisations [35]. Further, prior studies conducted in the DHS suggest a substantial amount of mistrust and lack of cooperation between physicians in the different settings in the DHS [36-38]. In addition to these clashing cultures, there also is a pervasive lack of information integration across settings and clinicians within the DHS. Accordingly, previous studies show that the coordination of care between GPs, hospitals, and municipalities has been insufficient [36,39]. Comparing hospitalisations for ACSCs within healthcare systems can serve as a surveillance mechanism to identify problems, but are not very precise in terms of identifying how to target the cause of that problem. However, together, these findings combined with previous studies on care coordination differences between DHS and KP [17], and on quality improvements within the KP system [34,40], suggest substantial opportunities to improve the quality and efficiency of care in Denmark for patients with chronic medical conditions, compared to the KP benchmark. In addition to providing a benchmark for potential quality improvement, the findings also suggest room for efficiency gains. Over the six year period from 2002 to 2007, the hospitalisation rates did decrease within DHS, but on average remained several fold greater in magnitude than the rates in KP, thus suggesting an upper bound for improvement. In other words, in 2007 alone, among the 32,001 persons hospitalised in Denmark for a preventable hospitalisation, 19,300 (60%) of them would not have been hospitalised, had the DHS rates been comparable to those in KP; there were 26,662 excess hospitalisations (i.e., 61% of the 43,521 observed hospitalisations in DHS in 2007 would not have occurred if the DHS rate was equal to that of KP's), and 5,599 excess readmissions (i.e., 61% of the 9,139 observed readmissions in DHS in 2007). Reallocating these resources from the hospital to preventing disease exacerbations in the outpatient setting could yield welfare gains for patients and their families, without requiring substantial new investments.

While additional research using individual level data on patient characteristics would improve the estimates of rates within each system, these longitudinal estimates within each system provide useful benchmarking information that can guide future reform efforts in the DHS as well as track the effects of any new reforms. Based on our results obvious areas for future reform efforts in the DHS may be improving the integration of services, improving structured care to persons with chronic conditions. However, it is critical to assess whether approaches from one healthcare system can be directly transferred to another system and whether major or minor changes should take place to obtain the desired effects [41]. Prior studies of implementation of technologies have shown that a technology, policy or function can be transformed in a new context and that the new context will influence how this approach is implemented and how it works [42]. Thus, caution must be exercised before transferring ideas or approaches used in KP to the Danish healthcare setting. Fireman et al. investigated savings resulting from the use of chronic care management programmes in KP. Actual cost savings were elusive, but programs could have sizable potential savings [34]. The study only focused on healthcare costs and savings. There is insufficient evidence that this approach will achieve the same improvements in the DHS; however it can be hypothesized that investing in efforts to improve the quality of chronic care by strengthening outpatient care settings in the DHS will lead to fewer preventable hospitalisations. Implementation of chronic care management programs in the DHS cannot be expected to create immediate savings in the healthcare budget, but the potential for improved quality of care and long term savings at the society level seems to be substantial.

External benchmarking can be a valuable tool for healthcare system reforms striving to improve performance as it can shed light on areas with potential for improvements and provide inspiration for how to reform organisation and delivery systems. As an example the study published by Feachem et al. in 2002 comparing cost and performance in Kaiser Permanente and the NHS was followed up by additional studies and played an important role in the decision about implementing chronic disease management approaches in the NHS [43].

## Conclusion

There are substantial differences between the DHS and KP in the rates of preventable hospitalisations, mean length of stay and readmission rates for ambulatory care sensitive, chronic medical conditions. These empirical benchmarking data suggest potential opportunities for improvements in chronic care quality and efficiency within the Danish healthcare system. Reductions in hospitalisations also could improve patient welfare and free considerable resources for use towards preventing disease exacerbations. However, the results of this study confirm, that the details of care organization and coordination between service providers including rehabilitation facilities and nursing homes are very important and coordination does not happen automatically even within very well established national healthcare systems like the DHS. These conclusions may therefore also apply for other healthcare systems like the NHS in the UK and the US Medicare system striving to improve the integration of care for persons with chronic conditions.

## Competing interests

The authors declare that they have no competing interests.

## Authors' contributions

MS designed the concept and conducts of the study, obtained, analysed and interpreted data, and drafted the manuscript. MP obtained, analysed, and interpreted data and commented on the manuscript. AF, JK and JH assisted in study design, data analysis, and data interpretation, and provided critical revision of the manuscript for important intellectual concepts. JS, AK, MR and FD assisted in study design and in interpreting the data and commented on the manuscript. All authors have approved the final submitted manuscript.

## Pre-publication history

The pre-publication history for this paper can be accessed here:

http://www.biomedcentral.com/1472-6963/11/347/prepub

## Supplementary Material

Additional file 1 **Ambulatory care sensitive conditions - ICD-9 and ICD-10 codes**. A list of the ICD-9-codes for ambulatory care sensitive conditions defined by the U.S. Agency for Healthcare Research and Quality and the used ICD-10 codes for the five selected conditions: angina (without procedures), chronic obstructive pulmonary disease (COPD), congestive heart failure (CHF), diabetes mellitus (DM), and hypertension (HTN). Click here for file

## References

[B1] Sox HC Learning from the health care systems of other countries Ann Intern Med 2008 148 78 79 18056653 10.7326/0003-4819-148-1-200801010-00197

[B2] Public Policy Commitee of the American College of Physicians Ginsburg JA Doherty RB Raiston JF Jr Senkeeto N Cooke M Achieving a high-performance health care system with universal access: what the United States can learn from other countries Ann Intern Med 2008 148 55 75 18056654 10.7326/0003-4819-148-1-200801010-00196

[B3] Schoen C Osborn R Doty MM Bishop M Peugh J Murukutla N Toward higher-performance health systems: adults' health care experiences in seven countries, 2007 Health Affairs 2007 26 w717 34 10.1377/hlthaff.26.6.w717 17978360

[B4] Billings J Zeitel L Lukomnik J Carey TS Blank AE Newman L Impact of socioeconomic status on hospital use in New York City Health Affairs 1993 12 162 173 8509018 10.1377/hlthaff.12.1.162

[B5] Bindman AB Grumbach K Osmond D Komaromy M Vranizan K Lurie N Preventable hospitalizations and access to health care JAMA 1995 274 305 311 10.1001/jama.1995.03530040033037 7609259

[B6] Bindman AB Chattopadhyay A Osmond DH Huen W Bacchetti P The impact of Medicaid managed care on hospitalizations for ambulatory care sensitive conditions Health Serv Res 2005 40 19 38 10.1111/j.1475-6773.2005.00340.x 15663700 PMC1361124

[B7] Epstein AM Revisiting readmissions--changing the incentives for shared accountability N Engl J Med 2009 360 1457 1459 10.1056/NEJMe0901006 19339727

[B8] Jencks SF Williams MV Coleman EA Rehospitalizations among patients in the Medicare fee-for-service program N Engl J Med 2009 360 1418 1428 10.1056/NEJMsa0803563 19339721

[B9] Peikes D Chen A Schore J Brown R Effects of care coordination on hospitalization, quality of care, and health care expenditures among Medicare beneficiaries: 15 randomized trials JAMA 2009 301 603 618 10.1001/jama.2009.126 19211468

[B10] Sharma G Fletcher KE Zhang D Kuo YF Freeman JL Goodwin JS Continuity of outpatient and inpatient care by primary care physicians for hospitalized older adults JAMA 2009 301 1671 1680 10.1001/jama.2009.517 19383958 PMC2771916

[B11] Caminal HJ Starfield B Sanchez RE Hermosilla PE Martin MM [Primary health care and hospitalizations in ambulatory care sensitive conditions in Catalonia] Rev Clin Esp 2001 201 501 507 11692404 10.1016/s0014-2565(01)70896-x

[B12] Dr Foster Intelligence Keeping people out of hospital 2006 London: Dr Foster

[B13] Rizza P Bianco A Pavia M Angelillo IF Preventable hospitalization and access to primary health care in an area of Southern Italy BMC Health Serv Res 2007 7 134 10.1186/1472-6963-7-134 17760976 PMC2045098

[B14] Saxena S George J Barber J Fitzpatrick J Majeed A Association of population and practice factors with potentially avoidable admission rates for chronic diseases in London: cross sectional analysis J R Soc Med 2006 99 81 89 10.1258/jrsm.99.2.81 16449782 PMC1360495

[B15] Strandberg-Larsen M Schiøtz M Frølich A Kaiser Permanente revisited - can European health care systems learn? Eurohealth 2007 14 24 26

[B16] Ham C York N Sutch S Shaw R Hospital bed utilisation in the NHS, Kaiser Permanente, and the US Medicare programme: analysis of routine data British Medical Journal 2003 327 1257 10.1136/bmj.327.7426.1257 14644968 PMC286244

[B17] Strandberg-Larsen M Schiotz ML Silver JD Frølich A Andersen JS Graetz I Is the Kaiser Permanente model superior in terms of clinical integration?: a comparative study of Kaiser Permanente, Northern California and the Danish Healthcare System BMC Health Services Research 2010 10 91 10.1186/1472-6963-10-91 20374667 PMC2907761

[B18] Feachem RG Sekhri NK White KL Getting more for their dollar: a comparison of the NHS with California's Kaiser Permanente BMJ 2002 324 135 141 10.1136/bmj.324.7330.135 11799029 PMC64512

[B19] Light D Dixon M Making the NHS more like Kaiser Permanente British Medical Journal 2004 328 763 765 10.1136/bmj.328.7442.763 15044296 PMC381335

[B20] Strandberg-Larsen M Schiøtz ML Frølich A Kaiser Permanente revisited - can european health care systems learn? Eurohealth 2007 13 24 26

[B21] Feachem RG Sekhri NK White KL Getting more for their dollar: a comparison of the NHS with California's Kaiser Permanente British Medical Journal 2002 324 135 141 10.1136/bmj.324.7330.135 11799029 PMC64512

[B22] Scott JT Rundall TG Vogt TM Hsu J Kaiser Permanente's experience of implementing an electronic medical record: a qualitative study British Medical Journal 2005 331 1313 1316 10.1136/bmj.38638.497477.68 16269467 PMC1298854

[B23] Strandberg-Larsen M Nielsen M Vallgårda S Krasnik A Vrangbaek K Mossialos E Denmark: Health system review Health Systems in Transition 2007 9 6

[B24] van der Zee J Kroneman MW Bismarck or Beveridge: a beauty contest between dinosaurs BMC Health Serv Res 2007 7 94 10.1186/1472-6963-7-94 17594476 PMC1934356

[B25] Frølich A Schiøtz ML Strandberg-Larsen M Hsu J Krasnik A Diderichsen F A retrospective Analysis of Health Systems in Denmark and Kaiser Permanente BMC Health Services Research 2008 8 252 10.1186/1472-6963-8-252 19077229 PMC2630928

[B26] AHRQ Quality Indicators - Guide to Prevention Indicators: Hospital Admission for Ambulatory Care Sensitive Conditions 2001 AHRQ Pub.No. 02-RO203. Rockville, MD: Agency for Healthcare Research and Quality

[B27] UCSF-Stanford Evidence-based Practice Center Refinement of the HCUP Quality Indicators 2001 Rockville, MD

[B28] Braveman P Schaaf VM Egerter S Bennett T Schecter W Insurance-related differences in the risk of ruptured appendix N Engl J Med 1994 331 444 449 10.1056/NEJM199408183310706 7880234

[B29] Song Y Skinner J Bynum J Sutherland J Wennberg JE Fisher ES Regional variations in diagnostic practices N Engl J Med 2010 363 45 53 10.1056/NEJMsa0910881 20463332 PMC2924574

[B30] Fisher ES Wennberg JE Stukel TA Skinner JS Sharp SM Freeman JL Associations among hospital capacity, utilization, and mortality of US Medicare beneficiaries, controlling for sociodemographic factors Health Serv Res 2000 34 1351 1362 10654835 PMC1089085

[B31] Williams EI Fitton F Factors affecting early unplanned readmission of elderly patients to hospital BMJ 1988 297 784 787 10.1136/bmj.297.6651.784 3142550 PMC1834386

[B32] Leng GC Walsh D Fowkes FGR Swainson CP Is the emergency readmission rate a valid outcome indicator? Quality in Health Care 1999 8 234 238 10.1136/qshc.8.4.234 10847885 PMC2483668

[B33] Victor CR Vetter NJ The early readmission of the elderly to hospital Age Ageing 1985 14 37 42 10.1093/ageing/14.1.37 4003176

[B34] Fireman B Bartlett J Selby J Can disease management reduce health care costs by improving quality? Health Affairs (Millwood) 2004 23 63 75 10.1377/hlthaff.23.6.63 15584100

[B35] Vinge S Buch MS Uhensigstmæssige indlæggelser - muligheder og perspektiver for kommunerne [in Danish Undesirable hospitalisations - posibilities and perspectives for the municipalities] 2007 Copenhagen: FOKUS DSI

[B36] Seemann J Antoft R Shared care: samspil og konflikt mellem kommune, praksislæge og sygehus: Aalborg kommunes demensudredningsmodel i praksis. [in Danish: Shared care: coordination and conflict between municipality, GP and hospital: The dementia diagnosing model of the municipality of Aalborg in practice] 2002 3. Aalborg: FLOS - Forskningscenter for Ledelse og Organisation i Sygehusvæsenet

[B37] Seemann J Jørgensen S, Hendriksen C, Falkesgaard N Barriers between sectors in Health care The Challenge of Chronic Diseases: can we do better? 2003 København: Klinisk Enhed for Sygdomsforebyggelse 37 42

[B38] Seemann J Løses sammenhængsproblemer med strukturdesign: hvad med kulturen? [in Danish: Are problems with coordination solved by structure design: what about the culture?] FLOSNyt 2004 Maj 8 10

[B39] Frolich A Host D Schnor H Norgaard A Ravn-Jensen C Borg E Integration of healthcare rehabilitation in chronic conditions Int J Integr Care 2010 10 e033 20216953 10.5334/ijic.507 PMC2834924

[B40] Yeh RW Sidney S Chandra M Sorel M Selby JV Go AS Population trends in the incidence and outcomes of acute myocardial infarction N Engl J Med 2010 362 2155 2165 10.1056/NEJMoa0908610 20558366

[B41] Schiotz ML Søgaard J Vallgarda S Krasnik A Internationale sammenligninger af sundhedssystemer [International comparisons of health systems] Ugeskr Laeger 2010 172 771 774 20211080

[B42] Latour B Woolgar S Laboratory life: social construction of scientific facts 1979 Beverly Hills: Sage Publications

[B43] Robinson R The managment of chonic diseases Health Policy Monitor 2004 04

