# Supplementary material for: Something is amiss in Denmark: A comparison of preventable hospitalisations and readmissions for chronic medical conditions in the Danish Healthcare system and Kaiser Permanente
Source: BMC Health Serv Res. 2011 Dec 22;11:347. doi: 10.1186/1472-6963-11-347 (PMC3258291; doi:10.1186/1472-6963-11-347)
Supplement: Additional file 1 — Ambulatory care sensitive conditions - ICD-9 and ICD-10 codes. A list of the ICD-9-codes for ambulatory care sensitive conditions defined by the U.S. Agency for Healthcare Research and Quality and the used ICD-10 codes for the five selected conditions: angina (without procedures), chronic obstructive pulmonary disease (COPD), congestive heart failure (CHF), diabetes mellitus (DM), and hypertension (HTN). [file 1472-6963-11-347-S1.PDF]

# Appendix I

Ambulatory care sensitive conditions – ICD-9 and ICD-10 codes

**Appendix 1 Ambulatory care sensitive conditions – ICD-9 and ICD-10 codes**

| ACS name | ICD-9 code | ICD-10 code | Description |
|---|---|---|---|
| **Angina** Exclude: All procedures: (01.0-86.99) | 4111 | I20.0-1, I20.8, I20.9, I24.0, I24.1, I24.8, I24.9 | Intermed coronary syndrome |
| | 41181 | I20.0-1, I20.8, I20.9, I24.0, I24.1, I24.8, I24.9 | Coronary occlsn w/o MI |
| | 41189 | I24.8 | Ac ischemic hrt dis nec |
| | 4130 | I20.9 | Angina decubitus |
| | 4131 | I20.0 | Prinzmetal angina |
| | 4139 | I20.9,I20,8 | Angina pectoris nec/nos |
| **COPD** | 4660 | J20 | Ac Bronchitis (qualify only if accompanied by secondary diagnosis of any of the other codes listed under COPD) |
| | 490 | J40.9/J40/J20.9 | Ac bronchitis nos (qualify only if accompanied by secondary diagnosis of any of the other codes listed under COPD) |
| | 4910 | J41.0 | Simple chr bronchitis |
| | 4911 | J41.1 | Mucopurul chr bronchitis |
| | 49120 | J44.9 | Obs chr brnc w/o act exa |
| | 49121 | J44.1 | Obs chr brnc w act exa |
| | 4918 | J42 | Chronic bronchitis nec |
| | 4919 | J42.9, J42 | Chronic bronchitis nos |
| | 4920 | J43.8, J43.9 | Emphysematous bleb |
| | 4928 | J43.8, J43.9 | Emphysema nec |
| | 494 | J47, J47.9,J47.9 | Bronchiectasis – oct00 |
| | 4940 | J47, J47.9,J47.9 | Bronchiectas w/o ac exac oct00 |
| | 4941 | J47, J47.9,J47.9 | Bronchiectasis w ac exac oct00 |
| | 496 | J44/J44.9/J44.8 | Chr airway obstruct nec |

**Appendix 1 (cont)**

| **ACS name** | **ICD-9 code** | **ICD-10 code** | **Description** |
|---|---|---|---|
| **Congestive heart failure rate** | | | |
| | 39891 | I09 | RHEUMATIC HEART FAILURE |
| | 40201 | I11.0 | MAL HYPERT HRT DIS W CHF |
| | 40211 | I11.0 | BENIGN HYP HRT DIS W CHF |
| | 40291 | I11.0 | HYPERTEN HEART DIS W CHF |
| | 40401 | I13.0 | MAL HYPER HRT/REN W CHF |
| | 40403 | I13.2 | MAL HYP HRT/REN W CHF&RF |
| | 40411 | I13.0 | BEN HYPER HRT/REN W CHF |
| | 40413 | I13.2 | BEN HYP HRT/REN W CHF&RF |
| | 40491 | I13.9 | HYPER HRT/REN NOS W CHF |
| | 40493 | I13.2 | HYP HT/REN NOS W CHF&RF |
| | 4280 | I50.0 | CONGESTIVE HEART FAILURE |
| | 4281 | I50.1 | LEFT HEART FAILURE |
| | 4289 | I50.9/I46.9 | HEART FAILURE NOS |
| Exclude: Cardiac procedures: | ICD-9 codes | ICD-10 code Procedure codes, not diagnosis codes | |
| | 3601 | KFNG02 (balloon), KFNG05 (stent) | PTCA-1 VESSEL W/O AGENT |
| | 3602 | KFNG02 (ballon), KFNG05 (stent) | PTCA-1 VESSEL WITH AGNT |
| | 3605 | KFNG02 (ballon), KFNG05 (stent) | PTCA-MULTIPLE VESSEL |
| | 3606 | KFNA, KFNC | INSERT CORONARY ART STENT OCT95- |
| | 3610 | KFNA, KFNC | AORTOCORONARY BYPASS NOS |
| | 3611 | KFNA, KFNC | AORTOCOR BYPAS-1 COR ART |
| | 3612 | KFNA, KFNC | AORTOCOR BYPAS-2 COR ART |

**Appendix 1 (cont)**

| ACS name | ICD-9 code | ICD-10 code | Description |
|---|---|---|---|
| Exclude: Cardiac procedures: (cont) | 3613 | KFNA, KFNC | AORTOCOR BYPAS-3 COR ART |
| | 3614 | KFNA, KFNC | AORTCOR BYPAS-4+ COR ART |
| | 3615 | KTF and KFW | 1 INT MAM-COR ART BYPASS |
| | 3616 | KTF and KFW | 2 INT MAM-COR ART BYPASS |
| | 3617 | KTF and KFW | ABD-CORON ART BYPASS OCT96- |
| | 3619 | KTF and KFW | HRT REVAS BYPS ANAS NEC |
| | 375 | KFQ | HEART TRANSPLANTATION |
| | 3770 | BFCA01 all numbers up to BFCA07 | INT INSERT PACEMAK LEAD |
| | 3771 | BFCA01 all numbers up to BFCA07 | INT INSERT LEAD IN VENT |
| | 3772 | BFCA01 all numbers up to BFCA07 | INT INSER LEAD ATRI-VENT |
| | 3773 | BFCA01 all numbers up to BFCA07 | INT INSER LEAD IN ATRIUM |
| | 3774 | BFCA01 all numbers up to BFCA07 | INT OR REPL LEAD EPICAR |
| | 3775 | BFCA01 all numbers up to BFCA07 | REVISION OF LEAD |
| | 3776 | BFCA01 all numbers up to BFCA07 | REPL TV ATRI-VENT LEAD |
| | 3777 | BFCA01 all numbers up to BFCA07 | REMOVAL OF LEAD W/O REPL |
| | 3778 | BFCA01 all numbers up to BFCA07 | INSER TEMP PACEMAKER SYS |
| | 3779 | BFCA01 all numbers up to BFCA07 | REVIS OR RELOCATE POCKET |

Appendix 1 (cont)

| ACS name | ICD-9 code | ICD-10 code | Description |
|---|---|---|---|
| **Diabetes with short-term complications** | 25010 | E11.1 | DM KETO T2, DM CONT |
| | 25011 | E10.1 | DM KETO T1, DM CONT |
| | 25012 | E11.1 | DM KETO T2, DM UNCONT |
| | 25013 | E10.1 | DM KETO T1, DM UNCONT |
| | 25020 | Cannot be classified by ICD-10 codes | DM W/ HYPROSM T2, DM CONT |
| | 25021 | Cannot be classified by ICD-10 codes | DM W/ HYPROSM T1, DM CONT |
| | 25022 | Cannot be classified by ICD-10 codes | DM W/ HYPROSM T2, DM UNCNT |
| | 25023 | Cannot be classified by ICD-10 codes | DM W/ HYPROSM T1, DM UNCNT |
| | 25030 | E11.0 | DM COMA NEC TYP II, DM CNT |
| | 25031 | E10.0 | DM COMA NEC T1, DM CONT |
| | 25032 | E11.0 | DM COMA NEC T2, DM UNCONT |
| | 25033 | E10.0 | DM COMA NEC T1, DM UNCONT |
| **Diabetes – uncontrolled (without short-term or long-term complications) admission rate** | 25002 | E11.9 | DM, T2, UNCONT |
| | 25003 | E10.9 | DM, T1, UNCONT |
| **Diabetes – long-term complications rate** | 25040 | E11.2 | DM RENAL COMP T2 CONT |
| | 25041 | E10.2 | DM RENAL COMP T1 CONT |
| | 25042 | E11.2 | DM RENAL COMP T2 UNCNT |

| | | | |
|---|---|---|---|
| | 25043 | E10.2 | DM RENAL COMP T1 UNCNT |
| | 25050 | E11.3 | DM EYE COMP T2 CONT |
| | 25051 | E10.3 | DM EYE COMP T1 CONT |
| | 25052 | E11.3 | DM EYE COMP T2 UNCNT |
| | 25053 | E10.3 | DM EYE COMP T1 UNCNT |
| | 25060 | E11.4 | DM NEURO COMP T2 CONT |
| | 25061 | E10.4 | DM NEURO COMP T1 CONT |
| | 25062 | E11.4 | DM NEURO COMP T2 UNCNT |

Appendix 1 (cont)

| ACS name | ICD-9 code | ICD-10 code | Description |
|---|---|---|---|
| **Diabetes – long-term complications rate (cont)** | 25063 | E10.4 | DM NEURO COMP T1 UNCNT |
| | 25070 | E11.5 | DM CIRCU DIS T2 CONT |
| | 25071 | E10.5 | DM CIRCU DIS T1 CONT |
| | 25072 | E11.5 | DM CIRCU DIS T2 UNCNT |
| | 25073 | E10.5 | DM CIRCU DIS T1 UNCNT |
| | 25080 | E11.6 | DM W COMP NEC T2 CONT |
| | 25081 | E10.6 | DM W COMP NEC T1 CONT |
| | 25082 | E11.6 | DM W COMP NEC T2 UNCNT |
| | 25083 | E10.6 | DM W COMP NEC T1 UNCNT |
| | 25090 | E11.6/E11.7/E11.8 | DM W COMPL NOS T2 CONT |
| | 25091 | E10.6/E10.7/E10.8 | DM W COMPL NOS T1 CONT |
| | 25092 | E11.6/E11.7/E11.8 | DM W COMPL NOS T2 UNCNT |
| | 25093 | E10.6/E10.7/E10.8 | DM W COMPL NOS T1 UNCNT |
| **Hypertension** | 4010 | Cannot be classified by ICD-10 codes | MALIGNANT HYPERTENSION |
| | 4019 | I10/I10.9 | HYPERTENSION NOS |
| | 40200 | I11.9 | MAL HYPERTEN HRT DIS W/OUT CHF |
| | 40210 | I11.9 | BEN HYPERTEN HRT DIS W/OUT CHF |
| | 40290 | I11.9 | HYPERTENSIVE HRT DIS W/OUT CHF |
| | 40300 | I12.9 | MAL HYPERT HRT DIS W/OUT RF |
| | 40310 | I12.9 | BENIGN HYP HRT DIS W/OUT RF |
| | 40390 | I12.9 | HYPERTEN HEART DIS W/OUT RF |
| | 40400 | I13.9 | MAL HYPER HRT/REN W/OUT CHF/RF |
| | 40410 | I13.9 | BEN HYPER HRT/REN W/OUT CHF/RF |
| | 40490 | I13.9 | HYPER HRT/REN NOS W/OUT CHF/RF |
| **Appendicitis** | 5400 | K35.0 | AC APPEND W PERITONITIS |

| | | | |
|---|---|---|---|
| **(non preventable)** | 5401 | K35.1 | ABSCESS OF APPENDIX |
| | 5409 | K35.9 | ACUTE APPENDICITIS NOS |
| | 541 | K37.9/K37 | APPENDICITIS NOS |
